# Pillars of Blood Pressure Management in Patients with Type 2 Diabetes Mellitus: Insights from Recent Trials and Emerging Perspectives

**DOI:** 10.3390/jcm14103269

**Published:** 2025-05-08

**Authors:** Călin Pop, Antoniu Octavian Petriş, Lavinia Pop, Liliana Elisabeta David

**Affiliations:** 1 County Emergency Hospital Baia Mare, 2nd Department Faculty of Nursing and Health Sciences, “Iuliu Hatieganu” University of Medicine and Pharmacy, 400337 Cluj Napoca, Romania; medicbm@yahoo.com; 2 Cardiology Clinic, “Grigore T. Popa” University of Medicine and Pharmacy, 700115 Iaşi, Romania; 3 “Sf. Spiridon” Clinical County Emergency Hospital, 700111 Iaşi, Romania; 4 Department of Metabolic Diseases, County Emergency Hospital, 430031 Baia Mare, Romania; laviniapop10@yahoo.com; 5 2nd Department of Internal Medicine, “Iuliu Hatieganu” University of Medicine and Pharmacy, 400337 Cluj Napoca, Romania; lilidavid2007@yahoo.com

**Keywords:** hypertension, type 2 diabetes mellitus, pharmacological treatment, blood pressure target, cardiovascular risk

## Abstract

Hypertension (HTN) is prevalent among patients with type 2 diabetes mellitus (T2DM), and both conditions are well-established risk factors for cardiovascular diseases (CVDs) and microvascular complications. To mitigate the risk of CVD, a comprehensive management approach for both blood pressure (BP) and glycaemic control is essential. The current therapeutic strategy should be structured around five pillars aimed at confirming HTN, establishing the 10-year CVD risk and its components and focusing on pharmacological treatment alongside lifestyle interventions to achieve BP targets. In clinical practice, the recommended BP target is 120–129/70–79 mmHg, while an optimal target of ≤120/70 mmHg is being explored under research conditions. Further, BP control should be re-evaluated in cases of resistant or uncontrolled HTN, in conjunction with antidiabetic therapies that have demonstrated cardiovascular and renal protective benefits. This five-pillar approach offers a comprehensive and evolving perspective on BP management in patients with T2DM, although certain aspects continue to be refined as new evidence emerges.

## 1. Introduction

The global prevalence of T2DM and HTN continues to rise, driven by aging populations, urbanization, and lifestyle factors such as poor diet, obesity, and physical inactivity. According to the International Diabetes Federation (IDF) Diabetes Atlas (2023 update), approximately 10.5% of the global adult population (537 million people) is living with diabetes, with over 90% of cases classified as T2DM. Projections suggest this number could reach 643 million by 2030 and 783 million by 2045 if current trends persist [1]. Similarly, HTN remains one of the leading risk factors for CVD worldwide [2]. The 2023 World Health Organization (WHO) report on hypertension estimates that 1 in 3 adults globally—about 1.28 billion people—are affected, with prevalence rates varying between 30% and 45% depending on the country and ethnic background [3]. Importantly, hypertension prevalence is significantly higher among individuals with diabetes. Recent studies indicate that up to 70–80% of people with T2DM have concomitant hypertension, highlighting a major intersection of cardiometabolic risk [4]. Data from the U.S. National Health and Nutrition Examination Survey (NHANES) 2017–2020 show that 74.4% of adults with diagnosed diabetes also had hypertension, a significant increase compared to the 57.3% figure reported in 2005–2008 [5]. In contrast, patients with type 1 diabetes continue to have a lower, though still elevated, HTN prevalence of around 30–40% [6]. These patients commonly exhibit clustering of cardiovascular risk factors associated with insulin resistance and lipid metabolism disturbances, contributing to a two- to three-fold increase in cardiovascular morbidity and mortality, compared to individuals without T2DM. Nevertheless, improved screening for T2DM and more effective management of cardiovascular risk factors—particularly BP—have contributed to a general decline in the incidence of CVD, including coronary artery disease (CAD), myocardial infarction (MI), heart failure (HF), stroke, and peripheral artery disease (PAD) [7]. Effective hypertension control in individuals with T2DM is critical to mitigating the increased risk of cardiovascular complications, improving long-term outcomes, and reducing the global healthcare burden.

## 2. Methods and Research Design

This narrative review systematically collected evidence from key randomized controlled trials (RCTs) and meta-analyses relevant to BP targets in patients with T2DM. Guidelines from the European Society of Cardiology (ESC), European Society of Hypertension (ESH), American Diabetes Association (ADA), and the American College of Cardiology/American Heart Association (ACC/AHA) were also examined, with a focus on updates post-2017. The search was conducted in PubMed and major scientific databases (Scopus, Web of Science) using combinations of keywords such as “hypertension”, “diabetes mellitus”, “blood pressure targets”, “cardiovascular outcomes”, and “guidelines”. Filters were applied to include articles published in English, clinical trials, meta-analyses, and guideline documents published primarily between 1995 and 2024. Particular attention was paid to foundational and high-impact RCT, as well as the international guidelines mentioned above. Studies were selected based on predefined inclusion criteria: focus on hypertensive patients with T2DM, use of explicit BP targets, and evaluation of cardiovascular or renal endpoints. Key trials were identified manually through citation tracking of seminal guideline publications post-2017. The collected studies were critically appraised to compare the efficacy and safety of different BP thresholds, particularly regarding cardiovascular and renal outcomes. Diagnostic criteria were standardized according to the 2017 ACC/AHA and 2024 ESC recommendations, integrating office-based, home, and ambulatory BP monitoring. Cardiovascular risk stratification utilized validated prediction models such as ASCVD (atherosclerotic cardiovascular disease) and SCORE2-Diabetes (risk assessment models to estimate the 10-year risk of cardiovascular disease in Europe), considering hypertension-mediated organ damage (HMOD), renal impairment, and other comorbidities. Findings were synthesized to outline optimal BP targets in T2DM patients, emphasizing the need for individualized management based on total cardiovascular risk, age, and treatment tolerability. Therapeutic approaches were reviewed, aligning with European and American guidelines, highlighting both non-pharmacologic interventions (e.g., lifestyle modifications) and pharmacologic therapies for hypertension and diabetes.

## 3. Target Blood Pressure in Hypertensive Patients with Type 2 Diabetes Mellitus

The optimal BP target for patients with T2DM remains mired in debate. Differences persist in the target BP used across clinical trials and recommendations by professional societies. Consequently, clinicians may encounter uncertainty when managing BP in diabetics. Historically, the diagnostic threshold for HTN was defined as office BP (OBP) ≥ 140/90 mmHg, with a treatment target of <140/90 mmHg for both the general and diabetic populations, before 2017 [8,9]. The clinical trials underpinning these recommendations include The UK Prospective Diabetes Study (UKPDS)-38, which showed that reducing BP to <150/85 mmHg lowered the risk of HF and microvascular complications; Hypertension Optimal Treatment (HOT), which demonstrated that a diastolic BP (DBP) ≤ 80 mmHg significantly reduced the risk of major cardiovascular (CV) events and mortality, compared to DBP ≤ 90 mmHg; The Action in Diabetes and Vascular Disease: preterAx and diamicroN-MR Controlled Evaluation trial (ADVANCE), which found that maintaining BP between 136/73 and 140/73 mmHg reduced all-cause mortality, CV death, and major macrovascular and microvascular events; The International Verapamil SR-Trandolapril Study (INVEST), which reported that a systolic BP (SBP) of approximately 131.2 mmHg in the intensive treatment group reduced adverse CV events, vis à vis SBP ≥ 140 mmHg; and the landmark trial ACCORD BP, BP–(Effects of intensive blood-pressure control in type 2 diabetes mellitus) which indicated that if SBP is <120 mmHg and DBP is <70 mmHg, there was no reduction in CV outcomes, but the risk of stroke was reduced by 41%, compared to SBP 130 to 140 mmHg (however, renal failure and serious adverse events increased with intensive BP control) [10,11,12,13,14]. Thus, the ACCORD BP trial, which aimed to assess the impact of intensive BP management in patients with T2DM, demonstrated a significant reduction in stroke incidence but not a corresponding benefit concerning cardiovascular mortality. Although intensive treatment led to better BP control and reduced the risk of adverse events like stroke, it was associated with an increased incidence of serious adverse events, such as hypotension, syncope, and electrolyte imbalances. This highlights the importance of individualizing treatment based on patient characteristics, comorbid conditions, and the potential for both short- and long-term adverse effects. As suggested, the target BP changed after 2017, when the ACC/AHA guidelines recommended initially that it should be <130/80 mmHg [15]. The importance of intensive BP control was also emphasized for both general adults and DM patients. However, supporting evidence for this came from only one landmark trial—the Systolic Blood Pressure Intervention Trial (SPRINT)—which excluded patients with DM [14,16]. The SPRINT enrolled 9361 patients aged ≥50 with HTN (SBP ≥ 130 mmHg) and at least one risk factor for heart disease [clinical or subclinical CVD, other than stroke, chronic kidney disease (CKD), with estimated glomerular filtration rate (eGFR) of 20–59 mL/min/1.73 m^2^, a Framingham Risk Score for 10-year cardiovascular disease risk ≥ 15%, age > 75], and followed up with the participants for a median of 3.26 years. The trial demonstrated that intensive BP control to SBP < 120 mmHg resulted in significant cardiovascular benefits in high-risk HTN patients, compared to routine BP control to <140 mmHg. The primary outcome [MI, acute coronary syndrome (ACS), stroke, HF, and CV death] was significantly lower in the intensive treatment arm vis à vis the standard management arm [5.2% vs. 6.8%, hazard ratio (HR) 0.75, 95% confidence interval (CI) 0.64–0.89; *p* < 0.0001]. These results contrast with those of the ACCORD trial among diabetics, where intensive BP lowering was not associated with improved CV outcomes. Secondary effects (such as syncope, hypotension, and accelerated reductions in GFR in patients without CKD at baseline) were more frequent in the intensive treatment arm [13]. In 2017, new results from a post hoc study among ACCORD DM patients with high CV risk and SPRINT criteria (2592, 54.8%) showed that reducing SBP to <120 mmHg (with a median achieved SBP of 120.1 mmHg) significantly lowered the risk of composite endpoints (nonfatal MI and stroke, any revascularization, HF and CV death), compared to standard control of SBP at <140 mmHg. Notably, a subgroup analysis suggested a greater treatment benefit in patients with HbA_1c_ ≤ 8.0%. Uncontrolled diabetes may diminish any potential benefit of intensive BP treatment. Like the SPRINT, there were more frequent active treatment-related adverse events [17]. In the absence of other randomized clinical trials (RCTs), several meta-analyses have attempted to clarify the optimal BP target in hypertensive DM patients. Overall, these meta-analyses suggest that targeting SBP between 130 and 140 mmHg and DBP between 70 and 80 mmHg improves CV outcomes [18,19,20]. However, since 2021, new evidence from three trials has shown a reduction in CVD risk in DM patients with BP targeted at <120/80 mmHg: STEP (Trial of Intensive Blood-Pressure Control in Older Patients with Hypertension), ESPRIT (Lowering systolic blood pressure to less than 120 mmHg versus less than 140 mmHg in patients with high cardiovascular risk with and without diabetes or previous stroke: an open-label, blinded-outcome, randomized trial), and the landmark BPROAD trial (Intensive Blood-Pressure Control in Patients with Type 2 Diabetes) [21,22,23]. The BPROAD trial specifically aimed to determine whether an intensive BP lowering strategy (SBP ≤ 120 mmHg) in hypertensive patients with T2DM was safe and reduced CVD events, compared to standard treatment targeting of SBP ≤ 140 mmHg. The primary composite outcome (nonfatal MI or stroke, HF rehospitalization, CV death) for intensive versus standard treatment was 1.65% vs. 2.09% per year (hazard ratio [HR] 0.79, 95% confidence interval [CI] 0.69–0.90), *p* < 0.001. Secondary events, such as hypotension and elevated potassium concentration, were slightly more frequent in patients treated intensively but could be easily managed through adjustments in pharmacological treatment. Trials like BPROAD/STEP were conducted among East Asian populations and may have limited generalizability. Table 1 summarizes the key trials that have explored the optimal BP target for patients with T2DM.

Therefore, the intensive lowering strategy is advantageous, and we must implement a more aggressive SBP target in hypertensive T2DM patients, as recommended in routine practice by the latest guidelines since 2018. These recommendations-ESC, ACC/AHA and ADA-established as a principal objective in hypertensive DM patients the evaluation of CV risk and the targeting of BP to predict CV outcomes: for instance, BP should be <130/80 mmHg if there is a high or very high CV risk, and recently, the ESC/2024 guidelines targeted BP at 120–129/70–79 mmHg in all patients, if feasible and tolerated [2,12,24,25,26,27]. The latest ESC guidelines rule out any reasons to have distinct BP treatment targets in patients with DM and recommend pharmacological treatment for those with confirmed HTN (office BP/OBP ≥ 140/90 mmHg) and for those with office BP ≥130/80 mmHg after a maximum of three months of lifestyle intervention to reduce CVD risk–Class I Level A recommendation [25]. A target BP ≤ 120/70 mmHg is promising based on the discussed emerging data, but current guidelines still recommend <130/80 mmHg–Table 2.

## 4. The Pillars of Blood Pressure Management in Patients with T2DM

**Figure 1:**
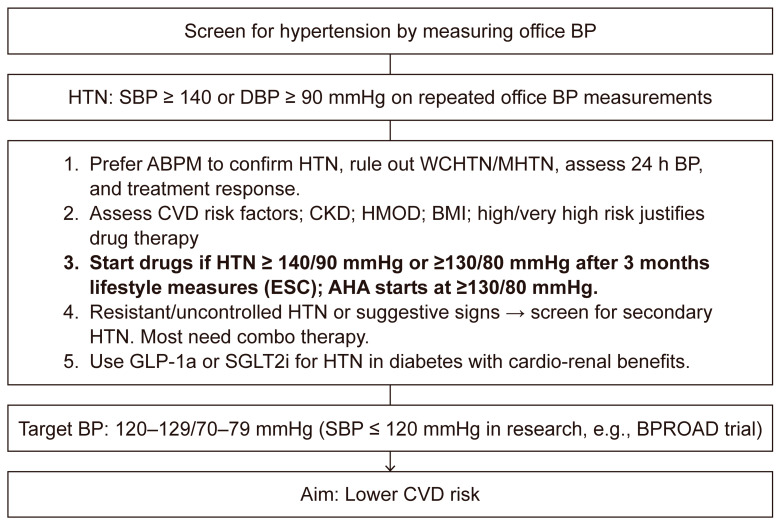
The five pillars approach in the management of HTN in T2DM patients. Legend: ABPM—automatic blood pressure measurements, BP-blood pressure, BMI—body mass index, CVD—cardiovascular, CKD—chronic kidney disease, HMOD—hypertension mediated organ damage, HTN—hypertension, MHTN—masked hypertension, WCHTN—white coat hypertension, h—hours, ESC—European Society of Cardiology, AHA—American Heart Association, SBP = systolic blood pressure, DBP—diastolic blood pressure, e.g.,—for example, BPROAD—Intensive Blood-Pressure Control in Patients with Type 2 Diabetes Trial, GLP1a—Glucagon-like-polypeptide 1 analogues, SGLT2i—Sodium-glucose-transporter 2 inhibitors, pts—patients.

The current therapeutic approach to managing BP in patients with T2DM should be structured around several pillars. These include confirming the diagnosis of HTN or elevated BP, establishing the 10-year CVD risk and its components, and focusing on implementing pharmacological treatment alongside lifestyle interventions to achieve the typical BP target of 120–129/70–79 mmHg in clinical practice. Additionally, BP control should be regularly reassessed—especially in cases of resistant or uncontrolled HTN—in conjunction with antidiabetic therapies with proven cardiovascular and renal protective benefits-Figure 1.

### 4.1. The First Pillar–Measuring and Confirming BP

The first pillar in the management of HTN or elevated BP in patients with T2DM is diagnosis. For confirmation, a single OBP measurement should be supplemented with additional BP assessment, preferably out-of-office or repeat office measurements, where out-of-office assessment is not feasible. Diabetics constitute a target group for home and ambulatory blood pressure monitoring (HBPM, ABPM) as diagnostic tools, due to their increased risk of stroke, target organ damage, and high CV morbidity and mortality. Conditions such as masked HTN (MHTN), no-dipper pattern, and nocturnal HTN necessitate ABPM for diagnosing HTN. All three patterns are frequently observed in diabetic patients (ranging from 30% to 73%) and are strongly correlated with an increase in albuminuria and left ventricular hypertrophy (LVH), as well as increased cardiovascular risk [28]. Evidence also indicates that BP values obtained through ABPM correlate better than OBP with target organ damage, particularly renal involvement, and cardiovascular events [29]. HBPM is also useful for detecting masked hypertension and is a better predictor of CV mortality and major adverse cardiovascular events (MACE) than OBP measurements [30]. Other important potential benefits of ABPM and HBPM include differentiation between sustained HTN and white-coat hypertension (WCHTN), assessing treatment response, improving treatment adherence, and establishing an appropriate therapeutic regimen to control nocturnal HTN, which could reduce CV risk [25]. Even when measured using the recommended standardized technique, routine OBP may be 5–10 mmHg higher than HBPM and ABPM values. According to the ESC 2024 guidelines, the updated definitions for elevated and hypertensive BP readings are as follows: elevated OBP 120/70–<140/90 mmHg, office HTN ≥ 140/90 mmHg; elevated BP at HBPM 120/70–<135/85 mmHg, HTN at HBPM ≥ 135/85 mmHg; elevated BP at 24 h ABPM 115/65–<130/80 mmHg, HTN at 24 h ABPM ≥ 130/80 mmHg [25]. Overall, the 2024 ESC guidelines recommend out-of-office BP measurement for diagnostic and therapeutic management purposes whenever possible (Class I, Level B), with many experts prescribing at least one ABPM measurement for hypertensive patients with T2DM [22,23,28,29,30].

### 4.2. The Second Pillar–Assessing CVD Risk and Comorbidities

The second pillar in HTN or elevated BP management in patients with T2DM involves conducting clinical and laboratory tests to detect traditional or newer cardiovascular risk factors, defining relevant comorbidities, and evaluating HMOD and CVD risk. These tasks should be performed systematically at the initial diagnosis of HTN or elevated BP and at least annually in all diabetics. Routine factors to identify include diabetes duration (HbA1c), smoking, dyslipidaemia (total cholesterol, LDL cholesterol, HDL and non-HDL cholesterol, and triglycerides), and tests for secondary HTN (hemoglobin). Additionally, the following should be evaluated: obesity/overweight, family history of premature CAD, a 12-lead ECG for heart HMOD and comorbidities (left atrial enlargement, LVF, atrial fibrillation/AF, CAD), and chronic kidney disease (CKD) by assessing creatinine blood level, eGFR, and the presence of albuminuria through urinalysis and urinary albumin-to-creatinine ratio. Hypertensive patients with T2DM often have multiple cardiovascular and renal comorbidities that increase CV risk; therefore, identifying overt CVD (e.g., CAD, MI, AF, HF, and PAD) or moderate-to-severe CKD or HMOD is crucial. This may warrant extended testing, especially to detect subclinical diseases. Additional assessments may include high-sensitivity troponins, NT-proBNP, echocardiography, abdominal, carotid, and peripheral artery ultrasound, fundoscopy, ankle-brachial index, and—in selected cases—coronary artery calcium via computed tomography. Most hypertensive patients with T2DM have very high or high CVD risk, and—specifically for individuals with elevated BP and T2DM below 60—the ASCVD risk score and SCORE2-Diabetes should be considered to identify those with moderate CVD risk (7.5% and <10% over 10 years) [31,32].

### 4.3. The Third Pillar–Lifestyle and Pharmacological Treatment

The central pillar in the management of HTN and elevated BP in T2DM patients focuses on the combination of lifestyle interventions and pharmacological treatment. The ESC 2024 guidelines recommend targeting BP in clinical practice at 120–129/70–79 mmHg, while the ACC/AHA 2017 guidelines initially proposed a target of <130/80 mmHg [15,25]. A more intensive target of ≤120/70 mmHg, as investigated in the BPROAD trial, shows promise but requires further validation, particularly in non-Chinese populations before it can be generalized to broader clinical practice [23]. Lifestyle interventions (non-pharmacological therapy) are recommended for HTN prevention in individuals with normal BP (<120/70 mmHg/ESC 2024; <120/80 mmHg/ACC/AHA 2017), for treatment in those with elevated BP (120/70 to 139/89 mmHg/ESC 2024; 120–129 and <80 mmHg/ACC/AHA 2017), and treatment in individuals with HTN (≥140/90 mmHg/ESC 2024; >130/80 mmHg/ACC/AHA 2017). These interventions are complementary approaches to reducing BP values and, in turn, CVD risk [15,25]. The lifestyle changes recommended include smoking cessation, weight reduction (e.g., ideal body mass index 20–25 kg/m^2^), a diet rich in fruits and vegetables (e.g., Mediterranean or DASH diet), restriction of free sugar consumption (e.g., maximum of 10% of energy intake), reduced intake of saturated and total fat, and limiting sodium intake to 2 g daily (e.g., Potassium-enriched salt comprising 75% sodium chloride and 25% potassium chloride is an option for individuals without advanced CKD). Additionally, aerobic physical activity (usually ≥30 min, 5–7 days/week) and reducing—or better still, avoiding—alcohol intake (e.g., maximum 100 g/week of pure alcohol), excess caffeine intake, or intake of drugs that can increase BP (e.g., glucocorticoids, sympathomimetics, nonsteroidal anti-inflammatory drugs) are advised. A supplementary argument for lifestyle interventions in hypertensive patients with T2DM is their beneficial effect on glycemic and lipid blood levels [24,25]. Based on available evidence, the ESC 2024 guidelines recommend that patients with DM and HTN (persistent OBP readings >140/90 mmHg) or SBP 130–139 mmHg after 3 months of lifestyle interventions should commence antihypertensive drug therapy [25]. The 2017 ACC/AHA guidelines recommend a lower threshold of ≥130/80 mmHg to initiate pharmacological treatment in these patients [15]. The major classes of antihypertensive drugs usually prescribed include angiotensin-converting enzyme inhibitors (ACEI), angiotensin receptor blockers (ARBs), dihydropyridine calcium channel blockers (CCBs), diuretics (thiazides and thiazide-like diuretics such as hydrochlorothiazide, chlorthalidone, and indapamide), beta-blockers (βB), and mineralocorticoid receptor antagonists (MRA).

Previous studies have demonstrated that ACE/ARBs and dihydropyridine CCBs—individually or in combination—are associated with reductions in CV and cerebrovascular outcomes, albuminuria, and CKD incidence and progression, particularly in hypertensive diabetics with microvascular complications [33,34,35]. Therefore, the extensive class of renin–angiotensin–aldosterone system (RAAS) blockers and dihydropyridine CCBs is recommended as first-line agents in the absence of contraindications. Diuretics are also an important class of agents used in the treatment of hypertensive diabetic patients. The prevention of HF is better achieved with diuretics as first-line agents or as add-on treatment while monitoring electrolytes and glycemic levels at the start of therapy [36]. βB agents have some metabolic effects (decreased HDL cholesterol, increased triglyceride levels, insulin resistance, weight gain, and masking of (hypoglycemia) that limit their use as first-line agents in diabetic patients. However, βB may be used as an add-on treatment in patients whose BP is not adequately controlled with other treatments, and in those with compelling indications for the use of βB, such as HF, CAD, or persistent tachycardia. MRAs such as the steroidal spironolactone (low dose-25 mg/day) or non-steroidal finerenone are effective for BP control and have renoprotective effects (reducing albuminuria and CKD progression) while lowering the risks of CVD events, especially in hypertensive patients with T2DM and CKD [37,38]. Hyperkalemia is the principal concern for patients treated with MRAs, particularly when combined with RAAS blockers as third- or fourth-line agents. Diuretics should be continued to prevent hyperkalemia when MRA are added. Other agents, such as alpha-blockers or centrally acting drugs, do not adversely affect the metabolic profile; however, they are reportedly less effective in preventing stroke and HF [39,40]. According to clinical practice, approximately 70% of hypertensive patients are not controlled by monotherapy and require an average of three drugs for adequate control [41]. Many hypertensives diabetic patients are treated with RAAS blockers, and current guidelines recommend dihydropyridine CCBs or diuretics as add-on therapy. According to the ACCOMPLISH trial, an ACEI combined with a dihydropyridine CCB (benazepril and amlodipine) is more effective in reducing the risks of stroke and CVD complications than a combination of ACEI and diuretic (benazepril and hydrochlorothiazide). Therefore, dihydropyridine CCBs are more appropriate than diuretics as second-line agents in hypertensive diabetic patients already treated with RAAS blockers [33]. Diuretics should be used in those requiring triple therapy unless there is a particular indication for using a different antihypertensive class, such as βB. MRAs are useful when HTN is resistant and when potassium is low. Antihypertensive treatment should be continued once the target BP has been achieved, as discontinuation is associated with an increased risk of combined cardiac, vascular, and stroke events [12,14].

### 4.4. The Fourth Pillar–Assessing Resistant or Uncontrolled HTN

Uncontrolled HTN refers to patients whose BP values remain above the target despite treatment, either due to ineffective treatment or lack of treatment initiation. Resistant hypertension (RH) is defined as BP values above the target despite the concurrent use of three antihypertensive drug classes, including diuretics [25]. The RIACE study, with a large cohort of 15,773 hypertensive patients with T2DM, found that RH (BP > 130/80 mmHg) was detected in 15% of the cohort, while uncontrolled HTN was present in 47%. Compared to those who achieved the target BP, patients with RH had a worse prognosis, with a two- to six-fold increase in the risk of cardiovascular and renal complications [42]. HBPM and ABPM should be employed to exclude pseudo-resistant HTN, including forms due to non-adherence to treatment, which accounts for one-quarter to two-thirds of all RH cases [25]. Confirmed RH should prompt the search for secondary causes of HTN, particularly those caused by diabetic nephropathy, renovascular etiology, and primary hyperaldosteronism, the latter having a 14% prevalence in one study [43]. To achieve the BP target for individuals with true RH or uncontrolled HTN after excluding potential curable secondary causes, synergistic and optimally dosed medicines should be prescribed. However, if BP remains uncontrolled under a maximally tolerated triple combination (RAAS blockers, CCB, and diuretic) therapy, and adherence has been assessed, the addition of spironolactone or eplerenone (when spironolactone is not tolerated) should be considered. Alternatives to MRAs in patients with advanced CKD or hyperkalemia include βB, particularly those with vasodilating properties (e.g., labetalol, carvedilol, or nebivolol). These patients should be referred to an expert center for appropriate work-up and treatment [15,25].

### 4.5. The Fifth Pillar–Diabetic Treatment with Protective Cardiovascular and Renal Effects, Effective for HTN Control

New medications that target different pathways in insulin and glucose metabolism have emerged in the last 20 years. Two such classes have demonstrated beneficial effects not only on glycemic levels but also on BP values, with significant preventive effects on cardiovascular and renal complications in diabetic patients. Sodium–glucose co-transporter 2 (SGLT2) inhibitors (e.g., canagliflozin, dapagliflozin, and empagliflozin) have shown a significant reduction in BP levels. A meta-analysis published in 2017 reported that using SGLT2 inhibitors resulted in a significant reduction in SBP of −2.46 mm Hg (95% confidence interval [CI]: −2.86 to −2.06 mm Hg) and a significant reduction in DBP of −1.46 mm Hg (95% CI: −1.82 to −1.09 mm Hg). The study also reported favorable effects on body weight and lipid profiles, indicating broader cardiometabolic benefits of SGLT2 inhibitors [44]. A new meta-analysis confirms the efficacy and safety of SGLT2 inhibitors on BP and glycemic control in hypertensive patients with T2DM [45]. The mechanism of BP reduction by SGLT2 inhibitors remains unclear; however, potential mechanisms include increased diuresis and natriuresis, nephron remodeling, RAAS inhibition, decreased arterial stiffness, and weight loss. Current guidelines recommend the early use of SGLT2 inhibitors in T2DM treatment, particularly for patients with established or high risk of atherosclerotic cardiovascular disease (ASCVD), HF, or CKD [46,47,48]. Glucagon-like peptide-1 analogs (GLP1a) induce significant weight loss in both diabetic and non-diabetic patients, potentially leading to improved BP control [49]. Like SGLT2 inhibitors, GLP1a reduces the risk of major adverse cardiovascular events (MACE), independently of their effect on glycemic control. GLP-1a leads to a reduction in SBP by 2 to 6 mm Hg, contributing to improved cardiovascular outcomes [50]. A 2025 meta-analysis showed greater BP reduction in overweight/obese patients using GLP-1a (SBP ↓3.37 mmHg, DBP ↓1.05 mmHg) [51]. GLP-1 receptor agonists are recommended for patients with T2DM and/or obesity, particularly when cardiovascular risk is elevated, to reduce the risk of MACE and support weight loss. They also offer modest benefits in BP reduction and may help delay CVD progression [52].

## 5. Areas of Uncertainty

Several key points of uncertainty include the following:

- Lack of consensus on BP targets: Different clinical trials and professional guidelines suggest varying BP targets for diabetic patients, with discrepancies between studies like ACCORD BP, SPRINT, and others. These trials yield conflicting results regarding the benefits of intensive BP control, especially concerning cardiovascular outcomes and adverse events.
- Conflicting evidence from trials: While some trials (e.g., SPRINT, BPROAD) support intensive BP control (<120 mmHg) for cardiovascular benefits, others (e.g., ACCORD BP) show no significant reduction in cardiovascular mortality, indicating that intensive control may not be universally beneficial for all T2DM patients.
- Potential risks of intensive BP control: Intensive BP lowering, particularly below 120 mmHg, has been linked to increased adverse events like hypotension, electrolyte imbalances, and kidney dysfunction, raising concerns about the safety of aggressive treatment strategies in certain patient groups.
- Generalizability of trial results: Some studies, such as the BPROAD trial, were conducted among specific populations (e.g., East Asians), and their findings may not be applicable to broader or more diverse groups, adding to the uncertainty regarding universal treatment guidelines.
- Changes in guidelines over time: There has been a shift in BP targets in clinical guidelines (e.g., from <140/90 mmHg to <130/80 mmHg), yet the supporting evidence is often based on a limited number of trials, leaving the question of an optimal target still open.

These uncertainties highlight the challenge of determining the best approach to BP management in diabetic patients, considering both the potential benefits and risks.

## 6. Strengths and Limitations

The proposed framework largely overlaps with existing consensus guidelines and prior systematic reviews. However, the originality lies in deeper interpretation, historical evolution, critical analysis of trial results, and proposing a didactic, oriented structure (pillars). Although guidelines mention these elements, organizing them as “pillars” for practical clinical workflow makes it easier for clinicians to systematize T2DM hypertension management. While guidelines endorse the use of antidiabetic agents with cardiovascular and renal benefits (e.g., SGLT2 inhibitors, GLP-1a), this synchronization with BP targets as part of structured BP management pillars is not formalized in most hypertension guidelines. This integrated perspective provides more holistic and context-specific management of BP in diverse patient populations with T2DM, particularly concerning personalized risk stratification and individualized treatment plans for patients with comorbidities.

## 7. Future Directions

Widespread validation and accessibility of HBPM devices are essential to improve the accurate diagnosis and management of all hypertensive patients. Several studies have been conducted in East Asian populations, whose general applicability may be limited. Therefore, further randomized clinical trials (RCTs) across other populations are needed to confirm the generalizability of an optimal target BP ≤ 120/70 mmHg achieved under research conditions. To reduce non-adherence and improve BP control, more RCTs that compare single-pill combination therapy with fixed doses (triple or quadruple combinations) versus multiple monotherapies and their effects on CVD outcomes in DM patients are also necessary. Artificial intelligence (AI)-enhanced diagnostic and therapeutic strategies may provide optimal approaches for general and diabetic hypertensive patients. Here are some examples of how AI can be useful in the field: analyzing risk factors (genetics, lifestyle, and comorbidities) to forecast the likelihood of developing hypertension; analyzing data to detect abnormal patterns, alerting clinicians or patients to issues before they become severe; optimizing drug selection and dosing to reduce trial-and-error prescribing; and finally, virtual assistants can educate patients, remind them to take medications, monitor lifestyle factors, and answer questions.

## 8. Conclusions

Current evidence supports intensive BP control in patients with T2DM and HTN to reduce cardiovascular and renal risks. Diabetics represent a key group for home and ambulatory BP monitoring due to the frequent occurrence of white-coat hypertension, masked hypertension, nocturnal and non-dipping patterns, uncontrolled HTN, and resistant HTN.

A target BP of 120–129/70–79 mmHg is recommended, emphasizing the need for individualized management to avoid overtreatment. Although promising, a target BP ≤120/70 based on the emerging data needs further studies to validate its application and long-term impact in diverse clinical settings.

Effective therapy requires a multidimensional approach: addressing cardiovascular risk factors, promoting lifestyle changes, and employing pharmacological strategies. RAAS blockers are preferred as first-line agents, with diuretics and CCBs as adjuncts when necessary. Additionally, SGLT2 inhibitors and GLP-1 receptor agonists, used for T2DM management, provide significant cardiovascular and renal protection and contribute to BP control.

The five-pillar framework provides a structured and holistic approach to BP management in patients with T2DM, ensuring that key clinical dimensions are addressed.

Proposed Key Takeaway:

- Intensive BP control (<130/80 mmHg or lower if tolerated) reduces CV events in T2DM but must be balanced against the risk of adverse effects.
- Individualized treatment based on CV risk, organ damage, and patient tolerance remains crucial.

## Figures and Tables

**Table 1:** Key trials that have explored the optimal blood pressure target for patients with T2DM.

Trial	Year	BP Target	Key Outcome
UKPDS 38 [10]	1998	<150/85 mmHg	Reduced stroke and microvascular complications; limited effect on major CV events.
HOT [11]	1998	≤80 mmHg (DBP)	Lower CV events in diabetic patients.
ADVANCE BP [12]	2008	<130/80 mmHg	Reduced major CV events and kidney disease progression.
INVEST [13]	2010	<130/85 mmHg vs. 130–139/<90 mmHg	No CV benefits with intensive control; increased mortality in some.
ACCORD BP [14]	2010	<120 mmHg vs. <140 mmHg	No reduction in major CV events; more adverse events.
ACCORD BP Substudy [15]	2011	<120 mmHg vs. <140 mmHg	Reduced stroke and HF hospitalizations; riskier in older patients.
SPRINT [16]	2015	<120 mmHg	Reduced CV events and mortality (non-T2DM).
STEP [21]	2019	<120 mmHg	Lowered stroke and HF risk; not specific to T2DM.
ESPRIT [22]	2024	<120 mmHg vs. <140 mmHg	Reduced CV death, MI, stroke, and HF hospitalization; greater benefit in T2DM.
BPROAD [23]	2024	<120 mmHg vs. <140 mmHg	Lower incidence of MACE, non-fatal stroke, non-fatal MI, HF hospitalization, cardiovascular death in overweight and diabetic patients.

BP—blood pressure, T2DM—type 2 diabtes mellitus, SBP—systolic blood pressure, DBP- diastolic blood pressure, HF—heart failure, MI—myocardial infarction, MACE—major adverse coronary events.

**Table 2:** Recommended target BP in patients with DM according to contemporary and older guidelines.

Guideline	Office BP Target	Key Notes
ESC 2024 [25]	120–129/70–79 mmHg	Same BP targets for DM and non-DM if feasible and tolerated.
ADA 2022 [24]	<140/90 mmHg (general); <130/80 mmHg (high ASCVD risk)	Individualized based on age, CVD, CKD, risk factors.
ESC Prevention 2021 [26]	120–130/<80 mmHg	SBP 130 mmHg if tolerated; DBP <80 mmHg.
ESC/ESH 2018 [2]	<130/80 mmHg	130–139/<80 mmHg for patients ≥65 years.
AHA/ACC 2017 [15]	<130/80 mmHg	Universal target for DM patients.
ADA 2017 [27]	<140/80 mmHg; <130/80 mmHg (high CVD risk)	Based on CVD risk.
JNC 8—2014 [8]	<140/90 mmHg	Same for general and diabetic populations.

BP—blood pressure, ASCVD—atherosclerotic cardiovascular disease, CVD—cardiovascular diseases, CKD—chronic kidney disease, DM—diabetes mellitus, DBP—diastolic blood pressure, SBP—systolic blood pressure, ACC—American College of Cardiology, ADA—American Diabetes Association, AHA—American Heart Association, ESC—European Society of Cardiology, ESH—European Society of Hypertension, IDF—International Diabetes Federation, JNC—Joint National Committee.

## Data Availability

Data sharing is not applicable to this article as no datasets were generated or analyzed during the current outline of advances in our field of expertise.
